# Sex and Gender Matters to the Heart

**DOI:** 10.3389/fcvm.2020.587888

**Published:** 2020-11-26

**Authors:** Hester Den Ruijter

**Affiliations:** Laboratory of Experimental Cardiology, University Medical Center Utrecht, Utrecht, Netherlands

**Keywords:** sex, gender, cardiovasccular medicine, women, men, heart

Although it is clear that sex and gender play a role in the development and progression of cardiovascular disease, the integration of sex and gender is a real challenge in the field of cardiovascular medicine. The specialty section Gender Cardiovascular Medicine is a new section of Frontiers in Cardiovascular Medicine dedicated to transformative science in the field of sex differences and publishes peer-reviewed articles across clinical, translational, and basic cardiovascular medicine.

Thus far, prominent scientific journals and scientific funding organizations such as the European Commission, the Canadian Institutes of Health Research, and the US National Institutes of Health made huge efforts to integrate sex and gender not only in clinical research, but also in translational and basic research with the goal to promote transparency, inclusion, and better science (1). Online modules, courses and manuscripts are now available on how to integrate sex and gender in scientific design, in analyses and in reporting. Also, for the (bio)medical curriculum, sex and gender integration is gaining more attention. With this shift toward better integration of sex and gender in science, we launch a Specialty Section in Frontiers in Cardiovascular Medicine to advance the field even further.

In the next few lines, I briefly describe the knowledge gaps regarding cardiovascular disease in women, and discuss the rationale for integration of sex and gender in cardiovascular medicine studies.

## Sex vs. Gender

Although the terms sex and gender are often confused, “sex” refers to biological and physiologic traits characterizing maleness and femaleness. “Gender” refers to the roles and behaviors of men, women, and the continuum of gender diversities in our society. As recently elegantly summarized (2) whereas biological sex likely plays a more substantial role in disease etiology, onset, and progression, gender can differentially affect disease risk, symptom recognition, disease manifestations, access to care, quality of care, and adherence to treatment recommendations.

In cardiovascular disease our historical view on cardiovascular patients is seen in many textbooks in which pictures of male patients dominate. Indeed, men have outnumbered women in the majority of the cardiovascular diseases, specifically at younger ages. This pattern seems to reverse during aging with more women than men becoming affected with cardiovascular diseases. In recent years, a trend is emerging of women contributing more to the population of heart diseases at younger ages (3, 4). In addition, the question arises if heart disease in women has not been underestimated due to (previously) unrecognized pathophysiology such as coronary microvascular disease and coronary spasms. Therefore, the accurate diagnosis of heart disease in women warrants urgent attention.

## Women in Clinical Studies, and Sex-Specific Pathologies

The cornerstone of evidence-based medicine are randomized clinical trials that mostly evaluate drug efficacy and safety profiles. Cardiovascular trials are known for low enrollment of women, mostly attributed to exclusion of patients of older age and the presence of co-morbidities, such as diabetes. Despite awareness of the importance of including women in clinical trials in cardiovascular disease, recent enrollment has not increased over time in trials that test novel therapeutic strategies for coronary artery disease and heart failure and remains at ~25% women (5). Neither has reporting of data stratified by sex improved over time in studies reporting on efficacy and adverse drug reactions in HF (6, 7).

Despite the low number of women in studies, and the lack of sex stratification, the awareness that sex and gender differences exist are starting to emerge. For instance, men more often suffer from obstructive CAD and HF with reduced ejection fraction and other atherosclerosis-driven diseases while women more often develop stable atherosclerotic disease with plaque erosion as feature, non-obstructive CAD and heart failure (HF) with preserved ejection fraction (8, 9). Non-obstructive CAD and HFpEF may no longer be considered benign with multiple studies showing a poor prognosis, high prevalence in women, and hypothesize on sex and gender-dependent mechanisms (10–15). Most striking sex differences in terms of high prevalence in either men or women are found within diseases that are far less common such as Brugada syndrome in men, and sudden coronary artery dissection and Tsako-tsubo cardiomyopathy in women (16, 17). Tsako-tsubo seems to be triggered by psychological stress and mimics the features of acute myocardial infarction. It gives rise to severe left ventricular dysfunction while the coronary arteries are open. The unknown etiology of these rare cardiovascular events in either men or women allows us to fully explore new (sex-biased) pathophysiological processes and new opportunities for drug development.

## It is not Better in Pre-clinical Studies

Similar trends of a male preference is also seen in pre-clinical studies, animal studies, and cellular studies (18). For animal studies, lack of female rodents is often attributed to the variable nature of the data caused by the reproductive cycle, yet this hypothesis has been studied in neuroscience and refuted (19, 20). High-throughput phenotype data comparing wildtype and mutant mice convincingly show that a large proportion of traits are influenced by sex (21). The current lack of sex-stratification in pre-clinical research may lead to unintended health risks. Drugs being retracted from the market due to unanticipated adverse drug reactions in women is an obvious health risk, but also for men this poses health risks, as their access to previously effective drugs are denied when taken from the market due to adverse drug reactions in women.

## Sex-Dependent Mechanisms and Manifestations of Cardiovascular Diseases

Genetics and hormones play an important role, with sex chromosomes functioning in all cells containing global gene regulators that are present in different doses between the sexes (22). The X chromosome is of interest in diseases that have a different prevalence between sexes. Inactivation of the X chromosome in women entails the random silencing of one of the two X chromosomes to compensate for the fact that men have only one. The X chromosomes contain genes involved in inflammation, and are perceived to contribute largely to the occurrence of autoimmune diseases that are highly female-specific (23). Auto-immune diseases also set the stage for accelerated atherosclerosis, and the role of X chromosome-related mechanisms needs to be deciphered in more detail. Furthermore, the Y chromosome has received increasing attention due to its perceived role in inheritance of CAD risk and atherosclerosis (24, 25). Also, mosaic loss of chromosome Y in leukocytes has been associated with many different diseases, among which atherosclerotic diseases (26).

For sex hormones, estrogen and androgens are known to influence the cardiovascular system in multiple ways. Yet, the potential protection of exogenous sex hormone therapy on coronary artery disease remains under debate, and is nowadays thought to be dependent on timing, duration and dose. This highlights the need for more rigorous research to understand when and how sex hormones affect cardiovascular health in women and men.

## Conclusions

For too long, researchers and clinicians have neglected sex and gender when reporting results related to cardiovascular disease. This is not necessarily due to sexism but rather to a lack of awareness that sex and gender can have such an impact on cardiovascular disease, whether in its development, progression, or treatment. We need to change this harmful misconception, and make research findings generalizable to everyone. Moreover, the potential of integrating sex and gender in cardiovascular studies is tremendous and can offer new perspectives on cardiovascular disease (mechanisms), inspire new research questions, and fill current knowledge gaps that society rightfully demands.

This new section comes at a time where we can leverage the momentum to call on our community to integrate sex and gender in cardiovascular studies to improve scientific quality. This will benefit the speed of translating research findings to the clinic, enhance equality between women and men, and thereby ultimately improve the cardiovascular health of both women and men.

## Author Contributions

The author confirms being the sole contributor of this work and has approved it for publication.

## Conflict of Interest

The author declares that the research was conducted in the absence of any commercial or financial relationships that could be construed as a potential conflict of interest.
